# A Wall Fragment of *Cutibacterium acnes* Preserves Junctional Integrity Altered by *Staphylococcus aureus* in an Ex Vivo Porcine Skin Model

**DOI:** 10.3390/pharmaceutics15041224

**Published:** 2023-04-12

**Authors:** Irene Magnifico, Angelica Perna, Marco Alfio Cutuli, Alessandro Medoro, Laura Pietrangelo, Antonio Guarnieri, Emanuele Foderà, Daniela Passarella, Noemi Venditti, Franca Vergalito, Giulio Petronio Petronio, Roberto Di Marco

**Affiliations:** 1 Department of Medicine and Health Science “V. Tiberio”, Università degli Studi del Molise, 8600 Campobasso, Italy; i.magnifico@studenti.unimol.it (I.M.); angelica.perna@unimol.it (A.P.); m.cutuli@studenti.unimol.it (M.A.C.); alessandro.medoro@unimol.it (A.M.); laura.pietrangelo@unimol.it (L.P.); a.guarnieri@studenti.unimol.it (A.G.); e.fodera@studenti.unimol.it (E.F.); daniela.passarella@unimol.it (D.P.); n.venditti@studenti.unimol.it (N.V.); roberto.dimarco@unimol.it (R.D.M.); 2 Department of Agricultural, Environmental and Food Sciences (DiAAA), Università degli Studi del Molise, 8600 Campobasso, Italy; franca.vergalito@unimol.it

**Keywords:** *Staphylococcus aureus*, skin dysbiosis, cutaneous diseases, bacteriotherapy, *Cutibacterium acnes*

## Abstract

(1) Background alteration of the skin microbiota, dysbiosis, causes skin barrier impairment resulting in disease development. *Staphylococcus aureus*, the main pathogen associated with dysbiosis, secretes several virulence factors, including α-toxin that damages tight junctions and compromises the integrity of the skin barrier. The use of members of the resident microbiota to restore the skin barrier, bacteriotherapy, represents a safe treatment for skin conditions among innovative options. The aim of this study is the evaluation of a wall fragment derived from a patented strain of *Cutibacterium acnes* DSM28251 (c40) alone and conjugated to a mucopolysaccharide carrier (HAc40) in counteracting *S. aureus* pathogenic action on two tight junction proteins (Claudin-1 and ZO-1) in an ex vivo porcine skin infection model. Methods: skin biopsies were infected with live *S. aureus* strains ATCC29213 and DSM20491. Tissue was pre-incubated or co-incubated with c40 and HAc40. (3) Results: c40 and HAc40 prevent and counteract Claudin-1 and Zo-1 damage (4) Conclusions: c40 and the functional ingredient HAc40 represent a potential non-pharmacological treatment of skin diseases associated with cutaneous dysbiosis of *S. aureus*. These findings offer numerous avenues for new research.

## 1. Introduction

The skin’s primary function is to provide a physical barrier that protects the underlying tissues from the external ambient. Due to a dense cellular network (the corneocytes) and a rich lipid matrix, the stratum corneum, the most superficial portion of the epidermis, guarantees this function [1]. 

Crucial components of skin defensive mechanisms are the tight junctions (TJs). They are barrier proteins that counteract pathogens, chemicals, and other external substances from body damage [2]. Several TJ proteins with diverse epidermal localisation patterns have been identified in human, porcine, murine, and canine skin [3,4,5,6,7]. For example, occludin (Ocln) or cingulin is a cell–cell junction restricted to the *stratum granulosum*. ZO-1 and Claudin-4 are found in the *stratum granulosum* and the upper *stratum spinosum*, whereas Claudin-1 is found in all the layers, including the *stratum corneum* [8]. 

A factor that critically modulates the skin’s barrier function and homeostasis is the colonising microbiota [9]. Under physiological conditions, the skin microbiota has a mutualistic relationship with the host. It contributes to cutaneous homeostasis by inflammatory response modulation and protection against disease-causing pathogens, regulating the skin’s pH balance. Moreover, this microbiota is also involved in producing vitamins, enzymes, and other biochemicals essential for skin health [10,11,12].

Among the most studied species belonging to the skin microbiota, *Malassezia*, *Cutibacterium*, *Staphylococcus*, and *Corynebacterium* spp. contribute to skin homeostasis through several mechanisms [13,14]. Indeed, their secretion of protease and lipase enzymes and free fatty acids are involved in skin lipidic-film surface breakdown [15]. Moreover, bacteriocin production prevents skin pathogen colonisation [16]. Furthermore, the quorum sensing importance in moulds and yeasts inhibition by indole-based mediators for Malassezia and other commensal bacteria has been demonstrated. 

In this scenario, an imbalance of these delicate relationships might influence the skin ecosystem, causing dysbiosis [12]. Dysbiosis is often driven by pathogenic microorganisms’ overgrowth and/or a decrease in the variety of microbiota composition [17], representing one of the main triggering factors in numerous skin disease pathogenesis [12]. Indeed, evidence shows that skin microbiota diversity is reduced in individuals with certain skin conditions [18]. For example, in atopic dermatitis patients, it has often been observed that the pathogen *Staphylococcus aureus* (*S. aureus*) increases in correspondence with a decrease of certain skin commensals levels, such as *S. epidermidis*, *S. hominis*, and *C. acnes* [19]. 

*S. aureus* is an opportunistic pathogen that, in eubiosis conditions, colonises the skin of healthy individuals. However, this pathogen can proliferate in inflammatory skin conditions, releasing virulence factors that promote bacterial adhesion and toxin synthesis, directly damaging tissue [20]. Besides being a skin commensal, 30% of individuals harbour *S. aureus* in the nose. Methicillin-resistant *S. aureus* (MRSA), which accounts for most of the antibiotic-resistant bacteria’s invasive infections, is carried by 2% of the population. Many severe and fatal diseases, such as bacteremia, endocarditis, pneumonia, osteomyelitis, skin infections, and sepsis, are sustained by *S. aureus* [21]. 

The principal *S. aureus* toxin involved in skin pathologies is the α-toxin, which causes the formation of heptameric pores on target cells, epithelial and endothelial breaches by adherens junctions breaking, and cytoskeleton impairment. In addition, δ-toxin or similar cytolytic peptides called phenol-soluble modulins (PSMs) may also activate mast cells. Lastly, exfoliative toxins can induce damage to desmosomes by cleaving desmoglein 1, leading to *Staphylococcus* scalded skin syndrome [22]. 

The lack of curative treatment for skin conditions (i.e., dysbiosis cutaneous disorders) has led to an increased interest in alternative and complementary therapies [23]. 

Recent insights into skin commensals have been applied to a new therapeutic strategy using live bacteria: bacteriotherapy. This therapy may hold the key to developing highly compliant treatments and medical cures for patients [24]. Therefore, as mentioned above, skin microorganisms work with their metabolites to maintain skin stability and interfere with pathogenic bacteria growth regulating the microbiota balance [25,26,27]. 

This therapeutic approach’s safety, effectiveness, and sustainability need to be further investigated. In particular, the administration of specific bacterial strains has been investigated as an exclusionary treatment strategy against pathogens associated with chronic skin disorders [28]. Besides using probiotic strains, the possibility of restoring skin eubiosis by applying viable or lysate-heat-killed or derived substances from commensal strains is gaining ground in treating skin disorders [29]. These findings encourage the potential use of specific cutaneous strains in restoring skin microbiome eubiosis. 

In addition to bacteriotherapy, other therapeutic strategies targeting α-toxin have already been investigated [30,31]. One antivirulence strategy for counteracting toxins is the use of antibodies. Passive immunisation would provide affected patients with immediate treatment. In contrast, active immunisation would require several boosters and a long period to achieve effective immune responses, ultimately lessening the severity of *S. aureus* infections [30]. 

In addition to anti-toxin antibodies, studies have shown the efficacy of synthetic nanoparticles resembling cell membranes, such as liposomes, in sequestering bacterial toxins and regulating alpha-hemolysin expression [32,33]. 

The antibacterial, antifungal, antiparasitic, and antiviral activities of Antimicrobial Peptides (AMPs) are highly diversified. In addition to a wide range of antibacterial properties, AMPs also have anti-toxin properties [34,35,36,37]. 

Compounds derived from natural products with anti-toxin capabilities represent another therapeutic strategy for treating *S. aureus* infections. While isorhamnetin and puerarin demonstrated inhibition of alfa-hemolysin expression, baicalin effectively disrupted α-toxin activity by preventing the formation of the toxin pore complex on the surface of the host cell [38,39,40,41].

However, these strategies have been tested in vitro or in vivo alone or in combination with antibiotics to attenuate α-toxin as a virulence factor in *S. aureus* infections and its resistant strains (MRSA) [30,31]. On the other hand, given the multifactorial etiopathogenesis of many skin disorders (i.e., atopic dermatitis), bacteriotherapy may provide an advantage over previous strategies. Indeed, in addition to direct action on virulence factors (i.e., α-toxin), it can ensure the restoration of cutaneous eubiosis [23,42,43].

Thus, the aim of the study is the evaluation of a wall fragment derived from a patented strain of *C. acnes* DSM28251 (donated by Aileens Pharma Srl) alone and conjugated to a mucopolysaccharide carrier (HAc40) in counteracting *S. aureus* pathogenic action on two tight junction proteins (Claudin-1 and ZO-1) in an ex vivo porcine skin infection model used as in vivo alternative for medical devices testing, drug skin permeation, and cosmetics studies [44]. 

## 2. Materials and Methods

### 2.1. Chemicals and Reagents

Tryptic soy broth was purchased from Biolife and prepared according to manufacturer instructions (30 g/L, pH 7.2). 

SuperFrost Plus slides used for histology and immunofluorescence assays were purchased from Thermo Scientific (Waltham, MA, USA). 

Anti-Claudin-1 Rabbit Polyclonal (13050-1-1AP) was purchased from PROTEINTECHTM (Chicago, IL, USA). Anti-ZO-1 Monoclonal (ZO-1-1A12) was purchased from INVITROGENTM (Waltham, MA, USA). All antibodies were used at a concentration of 1:100 in 1% NGS in PBS (1X). Goxms Alexa fluor plusTM 488 and goat anti-rabbit Alexa fluorTM 568 purchased from Thermo Scientific were used at a dilution of 1:1000 in 1% NGS in PBS (1X).

Hyaluronic acid (HA) with medium molecular weight (0.50 × 10^6^ DA) was provided by Xi’an Rongsheng Biotechnology Co., Ltd. (Xi’an, China), HA was dissolved in distilled water at 0.5 mg/mL. c40 purified bacterial fragments of *C. acnes* DSM28251 and HAc40 were used at 25 µg/mL and 0.5 mg/mL, respectively.

### 2.2. Bacterial Growth, Bacterial Suspensions, and Free Cells Supernatant (FCS) Preparation 

Cultivation of *Staphylococcus aureus* strains (ATCC29213 and DSM20491) was performed aerobically on a rotary shaker (120 rpm) at 37 °C overnight in tryptic soy broth. 

Bacterial suspensions were obtained by centrifugation at 7000× *g* for 10 min at 4 °C. The culture medium was removed, and the bacterial pellets were washed 2 times in PBS pH 7.4 and titrated to a final concentration of 10^8^ CFU/mL (OD600).

Free Cells Supernatant (FCS) from *S. aureus* DSM20491 was prepared as follows: The supernatant fraction was collected by centrifugation (6000× *g*, 15 min, 4 °C; and 10,000× *g*, 15 min, 4 °C). Then the supernatant was filtered through a 0.45 µm filter (to remove any remaining cells).

### 2.3. FCS Qualitative Analysis

Qualitative analysis of the FCSs’ content obtained from *S. aureus* DSM20491, ATCC29213, and culture medium alone was performed according to Lind et al. with some modifications [45]. Briefly, *S. aureus* strains were grown aerobically in 100 mL of tryptic soy broth, pH 7.2, on a rotary shaker (120 rpm) at 37 °C using a 4% inoculum from an exponentially developing culture. The bacteria were collected by centrifugation at 4 °C after 18 h (hours) (20 min at 16,000× *g*). The supernatants were filtered with 0.45 µm filters to remove all bacterial cells, mixed with solid ammonium sulfate (75% saturated), and left in a cold chamber for 2 h [31]. 

Samples were denatured in a 2X loading sample buffer (4% SDS, 50 mM Tris pH 6.8, 50 mM Tricine, 0.0005% Coomassie Brilliant Blue R250, 0.075% dithiothreitol, 12% glycerol) at 98 °C for 5 min, then separated on 12,5-10% Tris-Tricine SDS-polyacrylamide gel. The gel was stained in a staining solution (0.1% Coomassie Brilliant Blue R-250, 45% methanol, and 45% glacial acetic acid) for 1 h with gentle agitation. Subsequently, the gel was destained in a destaining solution. This solution was replenished several times until the background of the gel was fully destained. The molecular weight of the protein bands was determined by analysis with GelAnalyzer 19.1 software [46].

The FCS protein content was determined by spectrophotometric reading at 595 nm with ready-to-use Bradford reagents. 

For further experiments, *S. aureus* DSM20491 FCS was used at a final concentration of 15 µg/µL.

### 2.4. Explant Preparation

Porcine ear skin explant was performed according to the protocol provided by Hwang et al. Porcine ears were taken from animal carcasses immediately after sacrifice and kept at 4 °C until use (within 1 h after removal). The tissues were degreased, cut with a dermatome, cleaned with 70% ethanol, washed in PBS, and dissected to obtain about 6 mm wide biopsies. Tissues presenting with abnormalities, such as oedema, abrasion, or heavy streaks, were discarded [27]. Each biopsy was then positioned in 35 mm culture plates by placing the dermis into contact with the medium nutrients and the epidermis in the air–liquid interface. The maintenance DMEM was supplemented with 5% FBS, 2% P/S, gentamycin (50 mg/mL), amphotericin 1×, and dexamethasone (35 µg/mL). After 24 h, the tissue was cleaned, washed twice with PBS, and resuspended in a fresh medium for 24 h to remove contaminants [16]. Following 48 h, the tissue was prepared for infection. The tissue was washed thrice with PBS and then resuspended in 1 ml of DMEM supplemented with 5% FBS without antibiotics for 2 h. 

### 2.5. Experimental Design

#### 2.5.1. Preincubation 

The biopsies were pre-incubated with the test substances: c40, HA, and HAc40 at the concentration previously mentioned. The plates were incubated for 2 h at 37 °C with 10% CO_2_. After two hours, tissues were infected with *S. aureus* suspensions (10^6^ CFU/mL) and *S. aureus* DSM20491 FCS.

#### 2.5.2. Co-Incubation

Bacterial suspensions and FCSs were concomitantly administered with tested substances (HA, HAc40, and c40) on explanted tissues at the previously described concentrations for the co-incubation assay. Tissues were incubated for 24 or 48 h, washed thrice with PBS, and fixed in 10% buffered formalin until inclusion.

### 2.6. Hematoxylin and Eosin Stain

Briefly, sections embedded in paraffin from each specimen were cut at 5 mm, mounted on glass, and dried overnight at 37 °C. All sections were then deparaffinised in xylene, rehydrated through a graded series of alcohol, and washed in phosphate-buffered saline (PBS). Successively, the slides were stained for 1 min with hematoxylin. The staining tray was placed under a rather weak tap water jet for 10 min to wash away the excess hematoxylin. The slides were stained for 1 min with eosin solution and rinsed with water to remove the extra dye. The samples were dehydrated with ethanol solutions at decreasing concentrations, and successively, the slides were mounted with a xylene-based mounting medium and visualised with the Leica TM 5000p scanning system. Prevention of *Staphylococcus aureus* penetration into the stratum corneum was used as a benchmark to evaluate treatment effects with the test suspensions (HA, c40, and HAc40).

### 2.7. Immunofluorescence 

Briefly, sections embedded in paraffin from each specimen were cut at 5 mm, mounted on glass, and dried overnight at 37 °C. All sections were then deparaffinised in xylene, rehydrated through a graded series of alcohol, and washed in phosphate-buffered waline (PBS). PBS was used for all subsequent washes and antiserum dilution. Antigen unmasking was carried out through 4 washes of 2 minutes each with sodium citrate (pH 6.0) solution. Tissue sections were blocked with a solution of 10% NGS in PBS 1X for 1 h. Slides were then incubated at 4 °C overnight with anti-Claudin-1 Rabbit Polyclonal antibody (13050-1-1AP PROTEINTECHTM) or anti-Occludin monoclonal antibody (OC-3F10 INVITROGENTM) or anti-ZO-1 Monoclonal antibody (ZO-1-1A12 INVITROGENTM) all at a concentration of 1:100 1% NGS in PBS. After several washes (3 × 5 min) to remove excess antibody, the slides were incubated for 1 h with the secondary antibodies (Goxms Alexa fluor plusTM 488 antibody and goat anti-rabbit Alexa fluorTM 568 antibody) diluted 1:1000 in 1% NGS in PBS. After several washes (3 × 5 min), sections were incubated with 4′,6-diamidino-2-phenylindole (DAPI) for 5 min and washed again (3 × 5 min) before being incubated for 10 min with a Sudan black solution 70% ethanol. Slides were mounted and observed under a fluorescence microscope (C2/C2si confocal microscope—Nikon, Japan). Negative controls for each tissue section were prepared by substituting the primary antiserum with non-immune IgG. All slides were stained in a single batch for each experiment, receiving equal staining. As described by Selam et al., the immunofluorescence staining intensity was evaluated and classified as absent, weak, moderate, or intense [47]. 

### 2.8. Microscopic Observation

All images were captured with the DS-Qi2 camera (Nikon, Japan) of a C2/C2si confocal microscope (Nikon, Japan) mounted on a Ti2-U base with 20× optical zoom. NIS-Elements (Nikon, Japan) v.5.01 software was used for the analysis. All staining images were acquired and processed with the same settings, and representative areas were scanned. 

## 3. Results

### 3.1. FCS Qualitative Analysis

**Figure 1:**
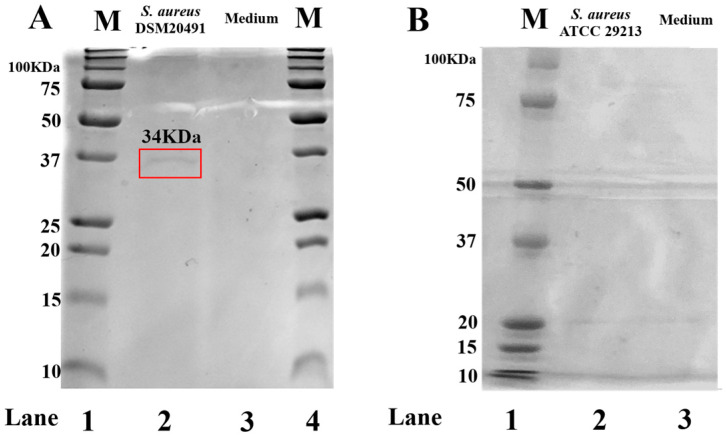
FCS qualitative analysis by SDS-PAGE. (**A**): Ladder (**lane 1**) Precision Plus Protein™ Unstained Protein Standards (BIORAD cat.no 1610363); *S. aureus* DSM4020491 (**lane 2**). (**B**): Ladder (**lane 1**) Precision Plus Protein™ Kaleidoscope™ Prestained Protein Standards (BIORAD cat.no 1610395) *S. aureus* ATCC29213 (**lane 2**). Samples were separated into 12% acrylamide gels. The bands’ molecular weight analysis was performed by GelAnalyzer 19.1 [46]. The culture medium (**lane 3**, Figure 1A,B) was used as a control. The gel was stained in Coomassie blue.

A band at 34 kDa corresponding to the molecular weight of the α-toxin monomer suggests the presence of α-toxin in *S. aureus* DSM20491 FCS (Figure 1A). As expected, qualitative analysis of the non-toxin-producing *S. aureus* strain ATCC29213 FCS confirmed the absence of the same band (Figure 1B). GelAnalyzer software analysis achieved the bands’ molecular weight [45]. 

### 3.2. Hyaluronic Acid, c40 Fragment Alone and Conjugated with Hyaluronic Acid on Hematoxylin-Eosin-Stained Sections of Animal Explants 

**Figure 2:**
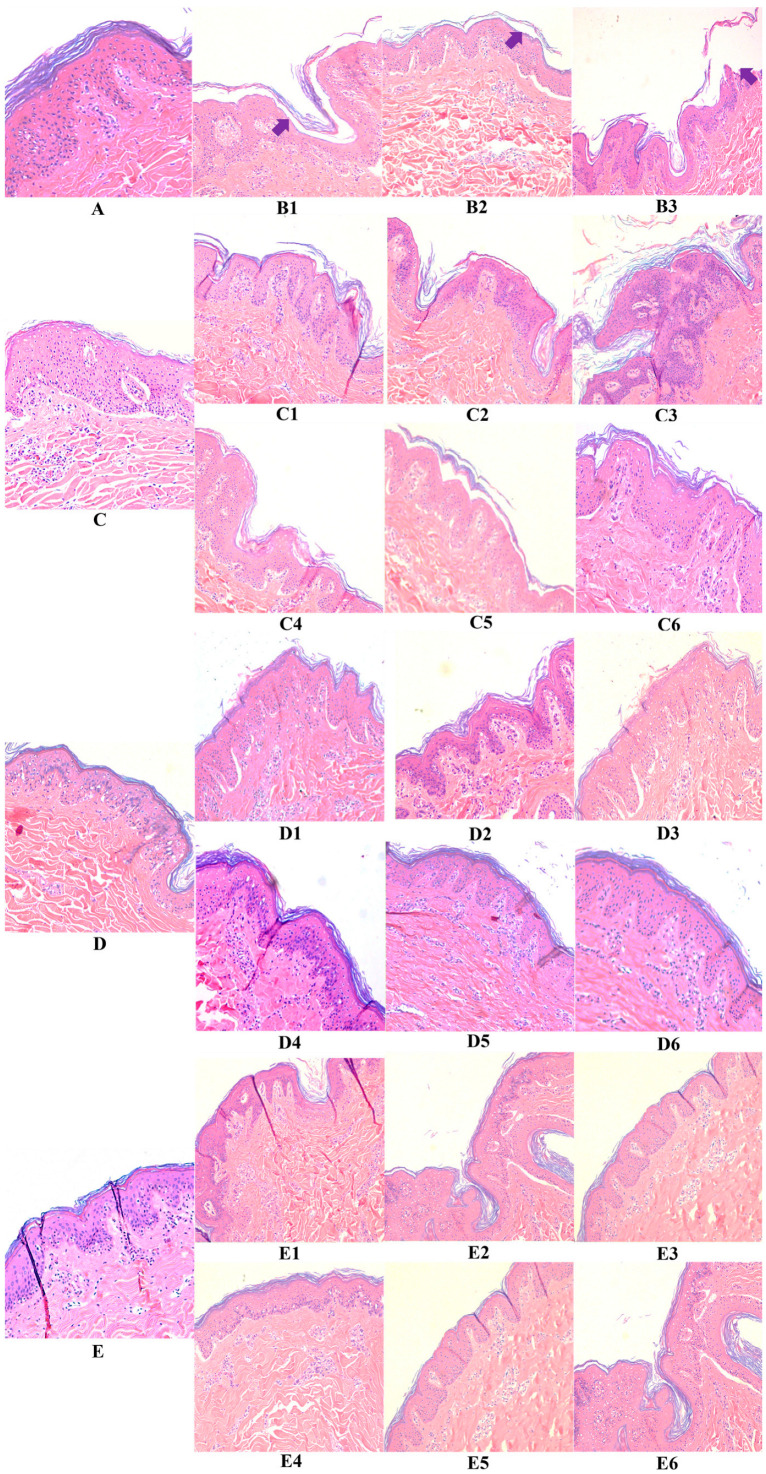
10× magnification eosin-hematoxylin stained tissues after 24 h infection with *Staphylococcus aureus* ATCC29213 and DSM20491 (**panels B1**,**B2**), FCS (**panel B3**), untreated (**panel A**) and treated (pre-incubated and coincubated) tissues with HA (**panels C1**–**C6**), c40 (**panels D1**–**D6**) and HAc40 (**panels E1**–**E6**).

**Figure 3:**
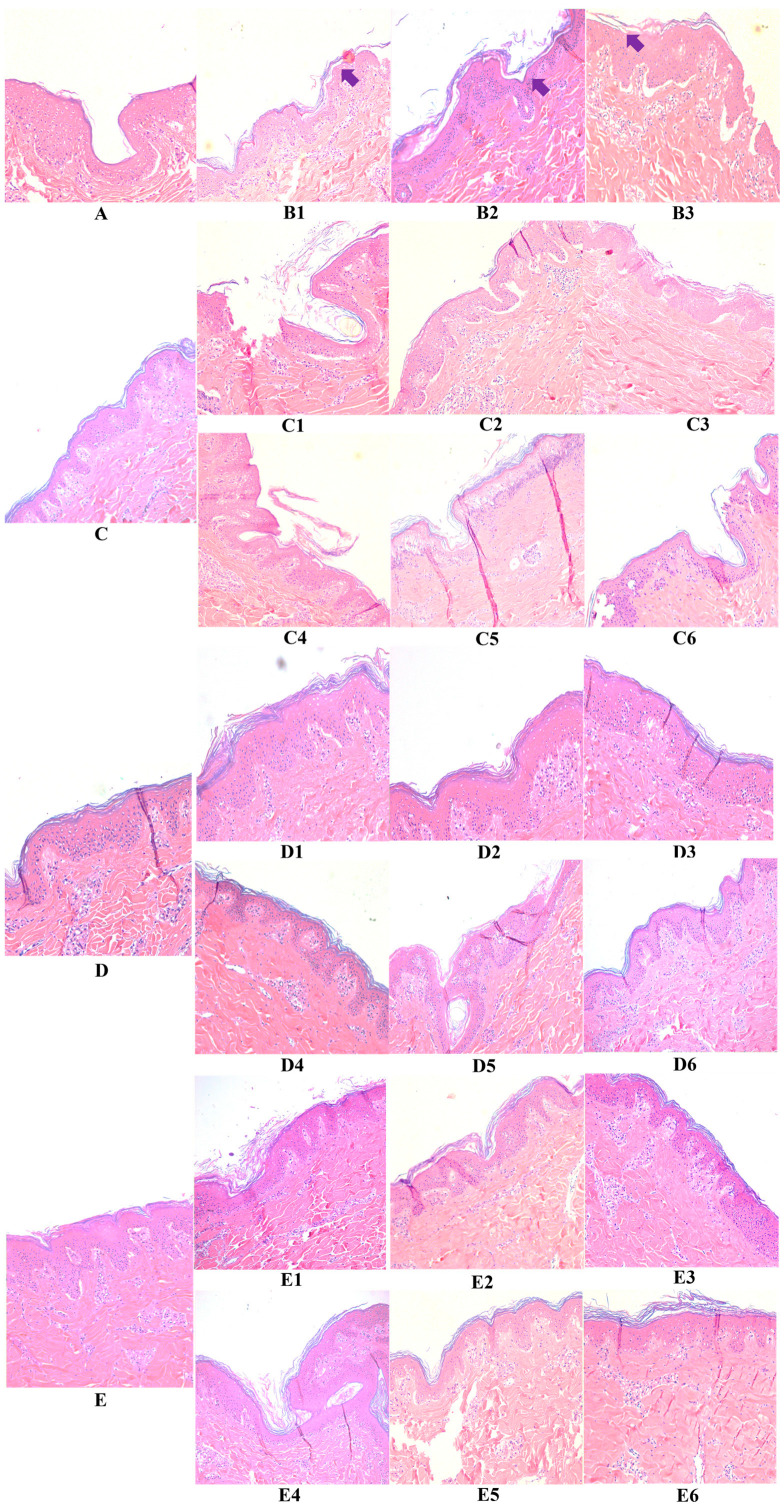
10× magnification eosin-hematoxylin stained tissues after 48 h infection with *Staphylococcus aureus* ATCC29213 and DSM20491 (**panels B1**,**B2**), FCS (**panel B3**), untreated (**panel A**) and treated (pre-incubated and coincubated) tissues with HA (panels **C1**–**C6**), c40 (**panels D1**–**D6**) and HAc40 (**panels E1**–**E6**).

Control tissues, i.e., “untreated” sample A, “hyaluronic acid” sample C, “fragment c40” sample D, and “HAc40” sample E, stained with eosin-hematoxylin (E&E) (Figure 2 and Figure 3) show a normal skin structure, free of morphological changes and with epidermis covered by cytokeratin. Conversely, 24 h (Figure 2) and 48 h (Figure 3) after *S. aureus* strains infection (ATCC29213 sample B1 and DSM20491 sample B2) along with FCS (sample B3), degeneration of keratinocytes as well as evident detachment of the stratum corneum were observed (full arrow). 

Similarly, pre-incubated (samples C1, C2, and C3) or co-incubated (samples C4, C5, and C6) tissues with HA and infected with the *S. aureus* strains or the FCS show marked signs of stratum corneum detachment, although intact. These findings suggested that HA alone could not protect from the pathogen- and FCS-induced damage. Similar results were obtained after 24 h (Figure 2) and 48 h of incubation (Figure 3).

In contrast, the morphology of c40 pre-incubated or co-incubated tissues (samples D1, D2, D3 and D4, D5, D6) and infected with the two bacterial strains or with the FCS shows a normal skin structure free of significant signs of degeneration (Figure 2). The same results were observed after the 48-h incubation period (Figure 3).

Similar findings were recorded for HAc40 pre-incubated (samples E1, E2, and E3) or co-incubated (E4, E5, and E6) tissues after 24 h (Figure 2) and 48 h of incubation (Figure 3).

### 3.3. c40, and HAc40 Activity on Animal Explant Sections Subjected to Immunofluorescence

**Figure 4:**
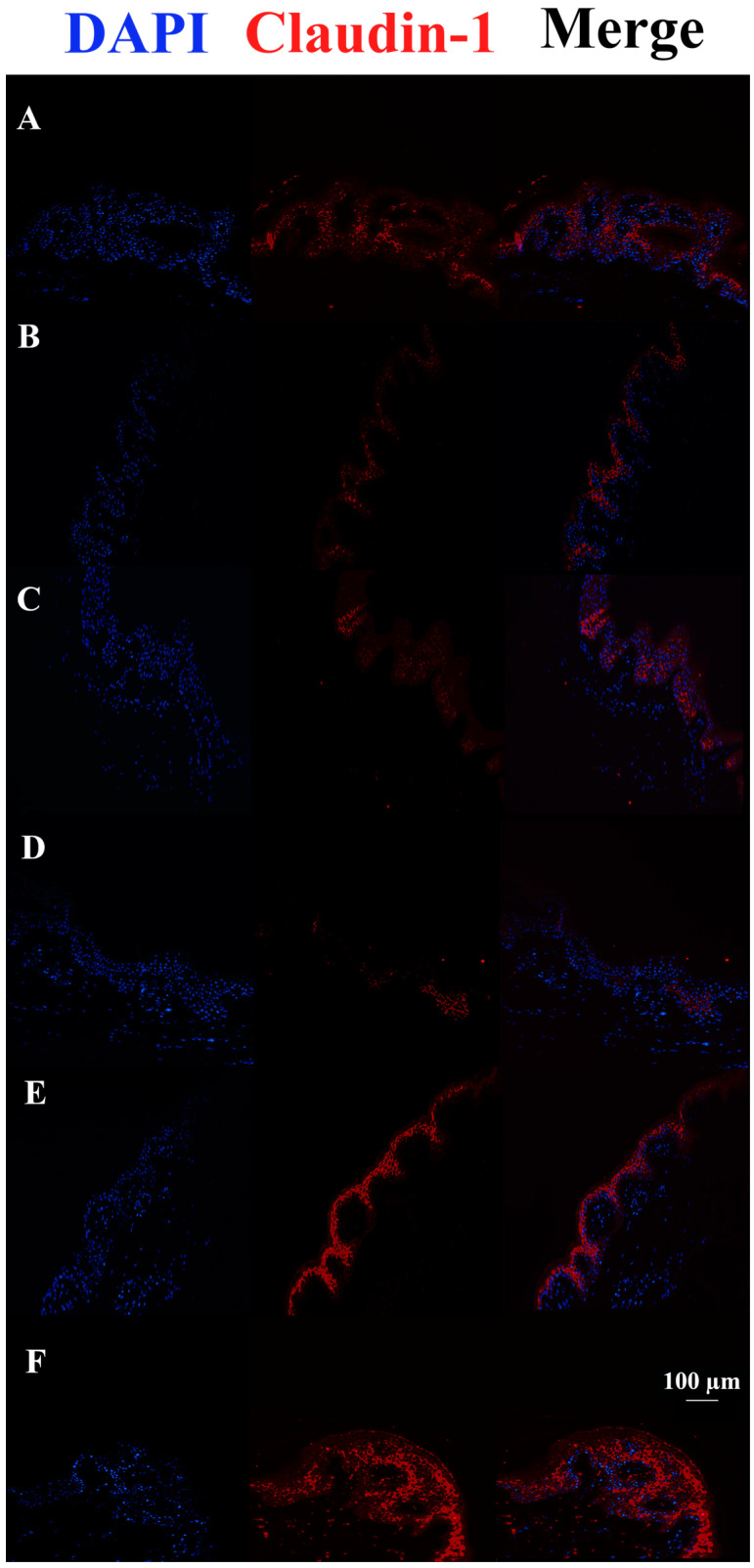
Claudin-1 immunofluorescence on animal explant sections infected with *S. aureus* ATCC29213 live bacteria (20× magnification). DAPI channel on the left side (461 nm blue); Anti-Claudin-1 1 antibody Alexa568-conjugated in the middle; images are shown on the right. Lane description. (**A**) Not infected tissues; (**B**) not treated infected tissues; (**C**) c40 pre-incubated infected tissues; (**D**) HAc40 pre-incubated infected tissues; (**E**) c40 co-incubated infected tissues; (**F**) Hac40 co-incubated infected tissues. The Claudin-1 signal is stronger in tissues treated with c40 and Hac40 than in infected tissues alone. Scale bar 100 µm.

After 48 h, Claudin-1 appears down-regulated in tissues infected with *S. aureus* ATCC29213 compared to control tissue. On the other hand, c40 and HAc40 treatments but not HAc40 preincubation were shown to have a more pronounced fluorescence signal than the control tissue (Figure 4).

**Figure 5:**
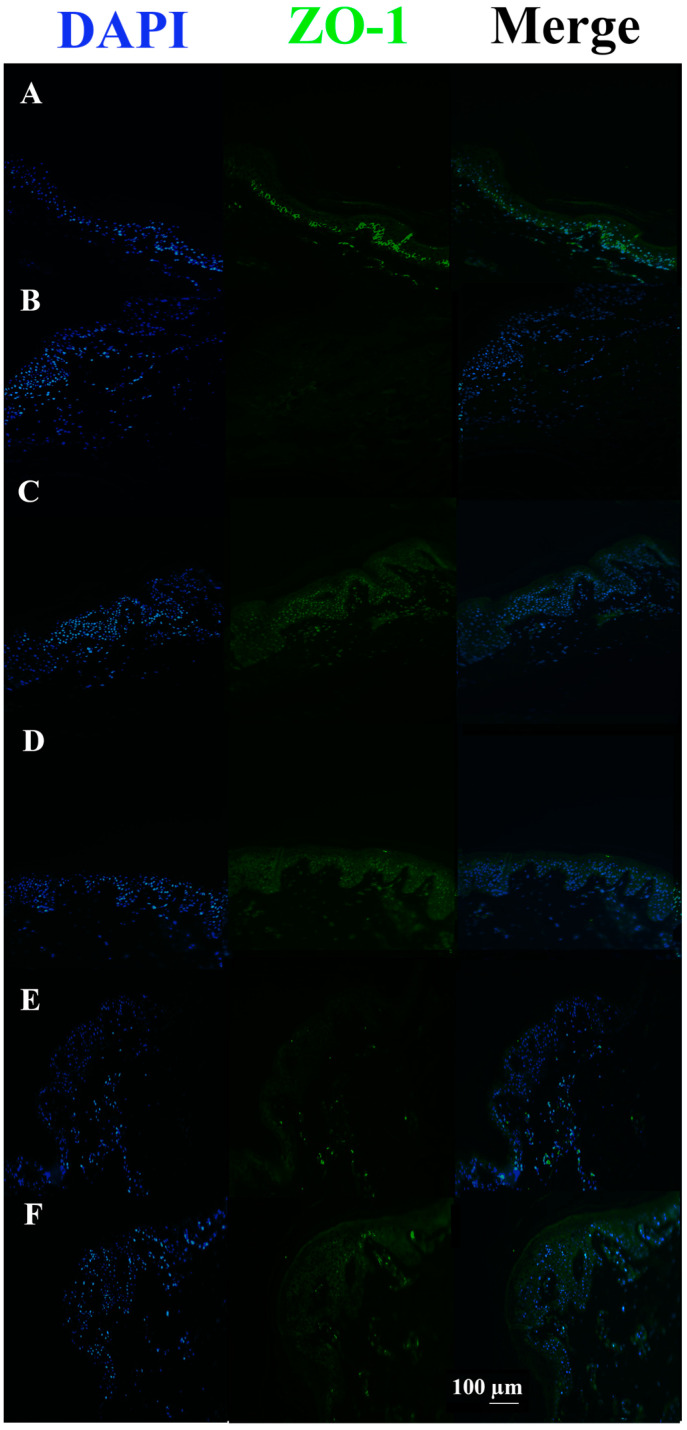
ZO-1 immunofluorescence on animal explant sections infected with *S. aureus* ATCC29213 live bacteria (20× magnification). DAPI channel (461 nm, blue) is shown on the left side; Anti-ZO-1 antibody Alexa488 nm-conjugated is in the middle; merge images are shown on the right. Lane description. (**A**) Not infected tissues; (**B**) not treated infected tissues; (**C**) c40 pre-incubated infected tissues; (**D**) Hac40 pre-incubated infected tissues; (**E**): c40 co-incubated infected tissues; (**F**) Hac40 co-incubated infected tissues. The ZO-1 signal in Hac40 co-incubated tissues was almost comparable to the untreated control. Scale bar 100 µm.

In *S. aureus* ATCCC29213 infected tissues, the ZO-1 signal was almost completely absent compared to the untreated control. Conversely, all infected tissues pre-incubated or co-incubated with c40 and Hac40 showed ZO-1 up-regulation, with a signal almost comparable to the control (Figure 5).

**Figure 6:**
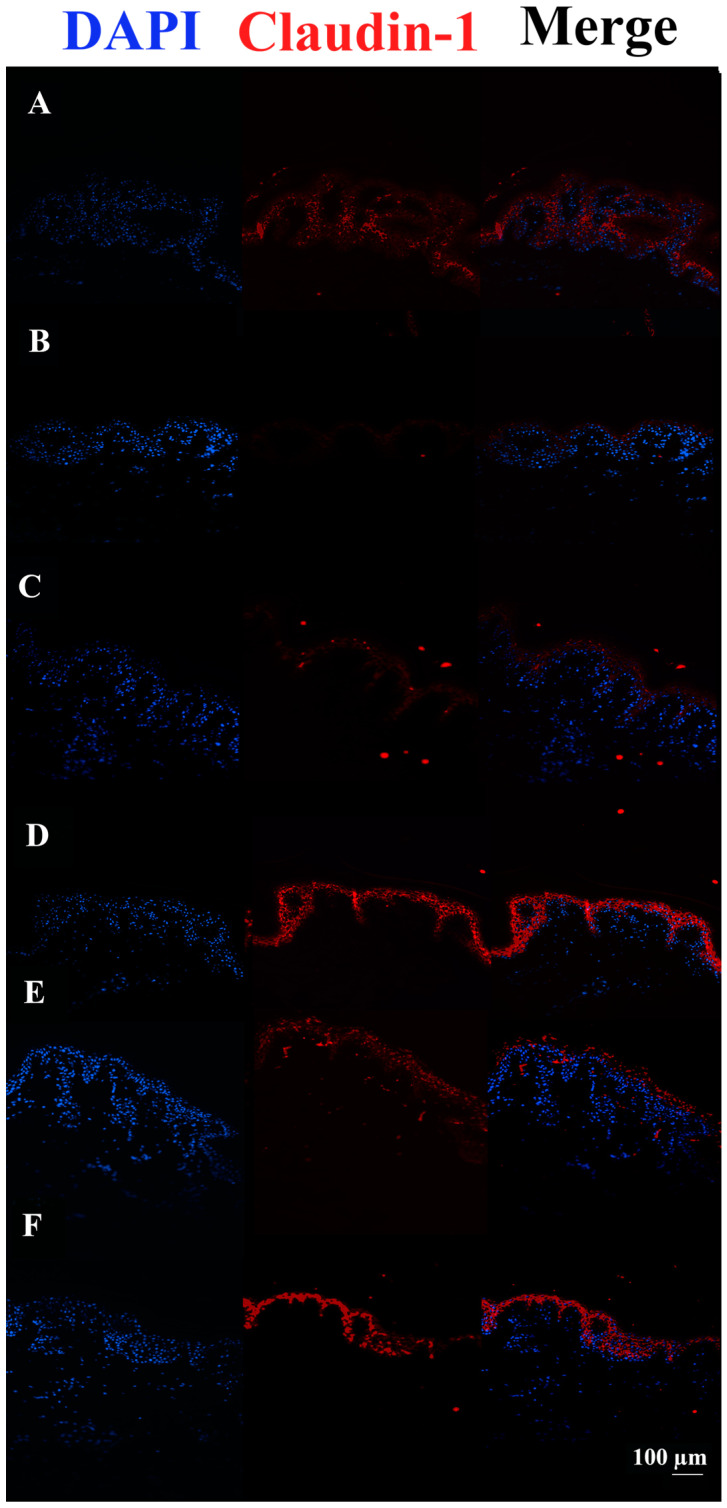
Claudin-1 immunofluorescence on animal explant sections infected with *S. aureus* DSM20491 live bacteria (20× magnification). DAPI channel (461 nm, blue) is shown on the left side; Anti-Claudin-1 antibody Alexa568-conjugated is in the middle; merge images are shown on the right. Lane description. (**A**) Not infected tissues; (**B**) not treated infected tissues; (**C**) c40 pre-incubated infected tissues; (**D**) Hac40 pre-incubated infected tissues; (**E**) c40 co-incubated infected tissues; (**F**) HAc40 co-incubated infected tissues. Tissues pretreated with c40 and HAc40 or co-incubated with HAc40 showed Claudin-1 up-regulation compared to untreated tissues. Scale bar 100 µm.

*S. aureus* DSM20491 live bacterial infection revealed a decrease in the Claudin-1 signal compared to not treated infected tissues. Conversely, all treatments increased the Claudin-1 signal, especially the Hac40 pre-incubation (Figure 6). 

**Figure 7:**
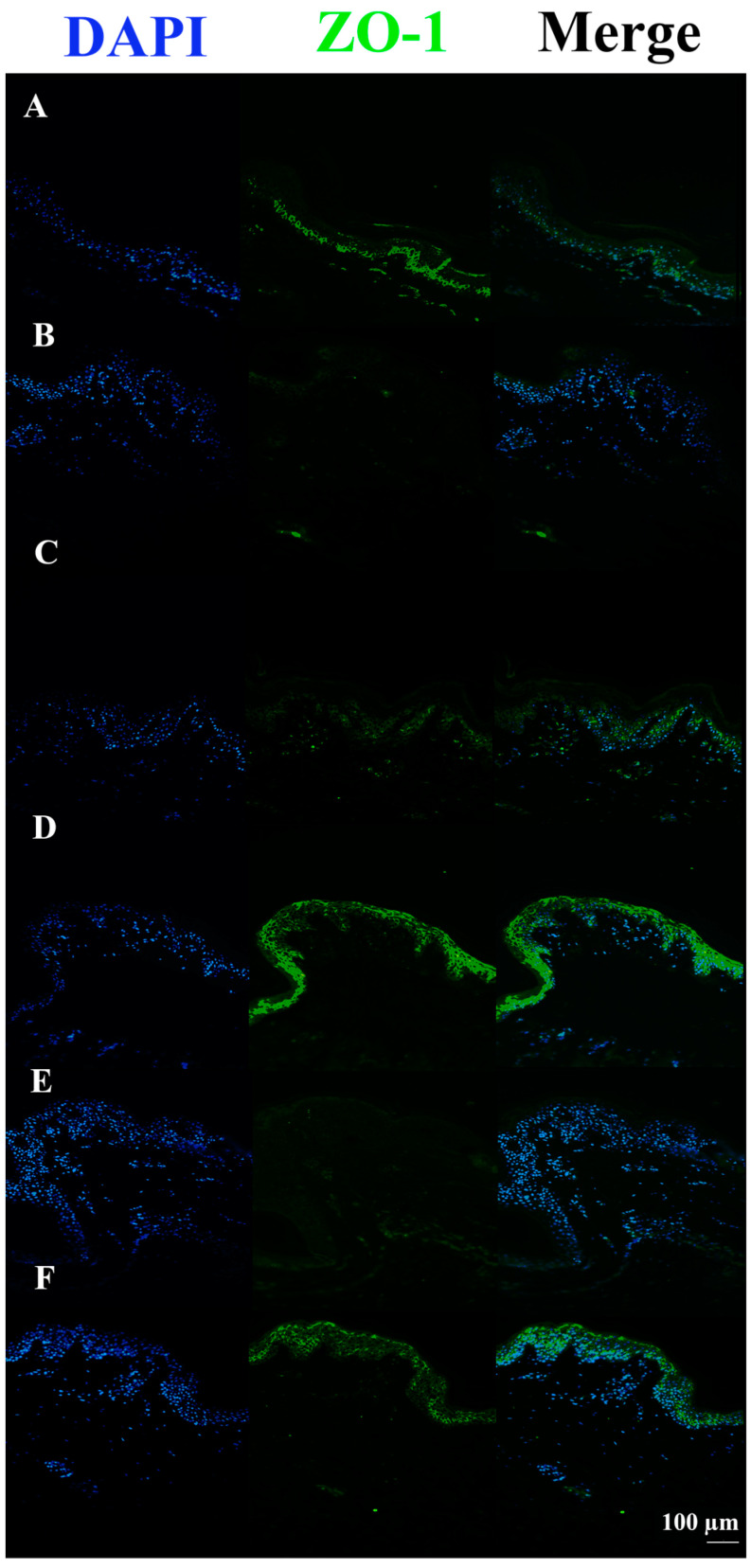
ZO-1 immunofluorescence on animal explant sections infected with *S. aureus* DSM20491 live bacteria (20× magnification). DAPI (461 nm, blue) is shown on the left side; Anti-ZO-1 antibody Alexa488 nm-conjugated is shown in the middle; merge images are shown on the right. Lane description. (**A**) Not infected tissues; (**B**) not treated infected tissues; (**C**) c40 pre-incubated infected tissues; (**D**) HAc40 pre-incubated infected tissues; (**E**) c40 co-incubated infected tissues; (**F**) HAc40 co-incubated infected tissues. c40 and HAc40 pre-incubated and HAc40 co-incubated tissues revealed a ZO-1 signal (green) almost comparable to untreated control. Scale bar 100 µm.

In *S. aureus* DSM20491 infected tissues, the ZO-1 signal was almost completely absent compared to untreated control tissue. Vice versa, all tissues infected with the pathogen but also pre-incubated or co-incubated with c40 and HAc40 (HAc40 co-incubated tissue excluded) showed an up-regulation in the ZO-1 signal, with an expression level comparable to the control tissue (Figure 7).

**Figure 8:**
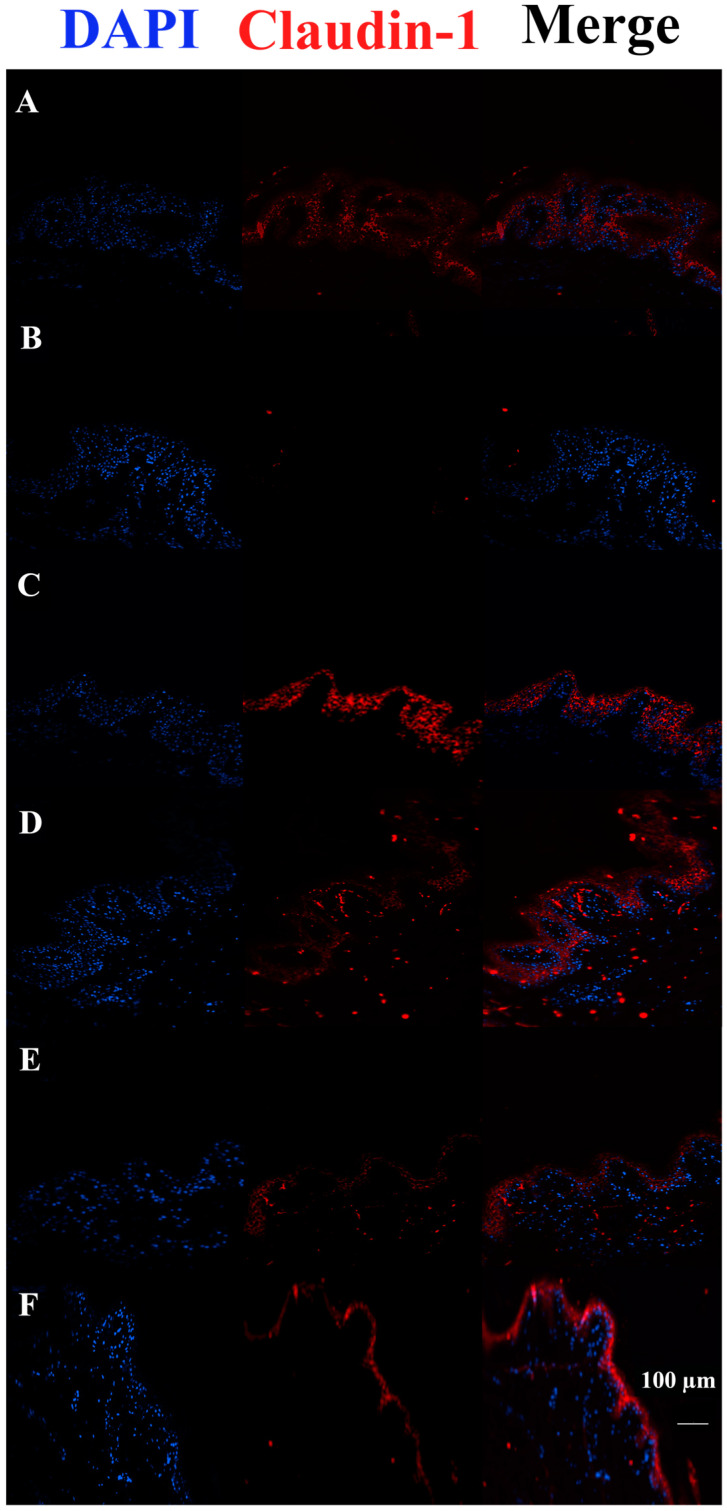
Claudin-1 immunofluorescence on animal explant sections infected with FCS processing (20× magnification). DAPI channel is shown on the left side (461 nm, blue); Anti-Claudin-1 antibody Alexa568-conjugated is shown in the middle; merge images are shown on the right. Lane description. (**A**) Not infected tissues; (**B**) not treated infected tissues; (**C**) c40 pre-incubated infected tissues; (**D**) HAc40 pre-incubated infected tissues; (**E**) c40 co-incubated infected tissues; (**F**) HAc40 co-incubated infected tissues. HAc40 pre-incubated and co-incubated tissues showed a Claudin-1 signal stronger compared to the FCS tissue alone. Scale bar 100 µm.

The Claudin-1 signal was completely absent in FCS-treated tissues. On the contrary, all treated tissues pre-incubated with HAc40 showed a stronger signal than controls. Remarkably, the Claudin-1 signal in c40 pre-incubated sections was comparable to the control (Figure 8).

**Figure 9:**
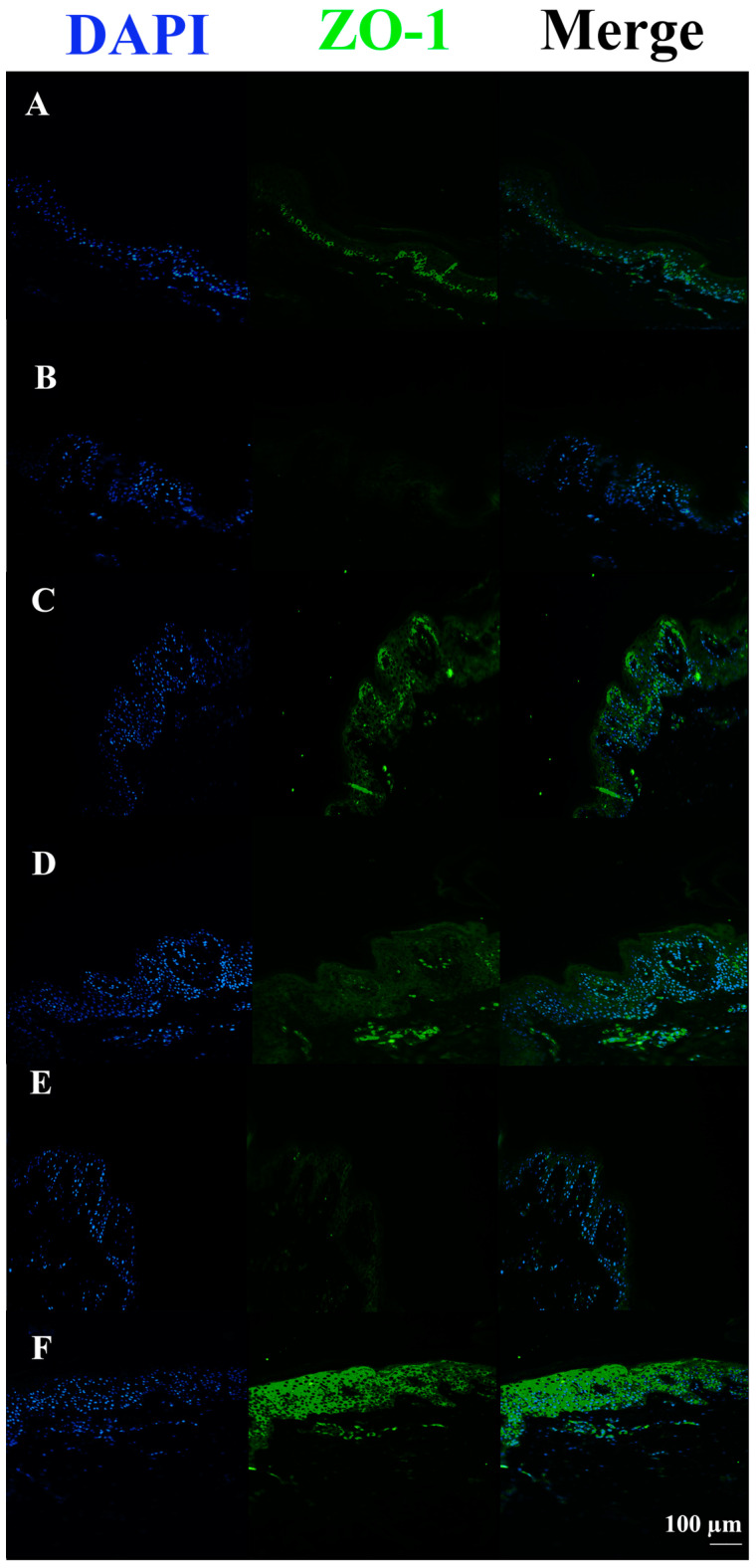
ZO-1 immunofluorescence on animal explant sections infected with FCS processing (20× magnification). DAPI (461 nm, blue) is shown on the left side; Anti-ZO-1 antibody Alexa488 nm-conjugated is shown in the middle; merge images are shown on the right. Lane description. (**A**) Not infected tissues; (**B**) not treated infected tissues; (**C**) c40 pre-incubated infected tissues; (**D**) HAc40 pre-incubated infected tissues; (**E**) c40 co-incubated infected tissues; (**F**): HAc40 co-incubated infected tissues. The ZO-1 (FITC) signal in Hac40 pre-incubated and c40 co-incubated tissues was almost comparable to the untreated control. Scale bar 100 µm.

Moreover, the ZO-1 signal was almost completely absent in tissues treated with FCS compared to the untreated control. All FCS-treated pre-incubated tissues showed stronger signals, almost comparable to the controls. Among these, HAc40-treated tissue revealed a ZO-1 signal comparable to the control (Figure 9).

## 4. Discussion

The investigation of bacteria’s therapeutic use in alleviating certain human diseases has gained popularity in recent years. Probiotic bacteria are known to bring numerous benefits to the human gut, such as epithelium repair, intestinal barrier function improvement, and immune response regulation [48]. 

The use of topical bacteriotherapy was first proposed for acne and seborrhea treatment back in 1912 when the efficacy of *L. bulgaricus* in improving symptomatology was demonstrated [49]. Recent studies have shown that probiotics and their derivatives can positively impact skin diseases [50]. For instance, experiments with lysates of *L. rhamnosus* GG (LGG) have shown that these products strengthen tight junctions and promote keratinocyte proliferation and migration [51]. 

In addition to well-known probiotic bacteria, studies on the possible therapeutic applications of the microorganisms belonging to the skin microbiota have been increasing in recent years [18,52]. 

Several pieces of evidence have proven that these microorganisms contribute to skin homeostasis by defending host cells against the proliferation of pathogenic microorganisms (such as *S. aureus*) and promoting skin barrier function [25,53]. 

Although the role of *C. acnes* species in skin health and disease is still being elucidated, it is now understood that there are substantial variations among strains, some of which have been demonstrated to have protective or even symbiotic properties [54]. This microorganism can regulate skin homeostasis by inhibiting pathogenic species through the secretion of specific bacteriocins or competition for nutrients [55].

In addition, *C. acnes* is able to secrete short-chain fatty acids (SCFAs) that are metabolised by the gut microbiome and have been shown to contribute to maintaining the colonic epithelium barrier by modulating tight epithelial junctions [29]. Furthermore, it colonises specific niches preventing the proliferation of pathogens. It has also been observed to modulate the immune response and oxidative stress mitigation [56]. The safety of topical treatment with a mixture of probiotic strains from the skin commensal *C. acnes* in patients with acne has been demonstrated recently by Karoglan et al. [25]. 

The main *S. aureus* virulence factors implicated in AD are a series of superantigens (adhesins and exotoxins) that mediate bacterial invasion and spread. These superantigens can alter skin barrier function, the microbiome composition, and the host immune response [57,58,59]. Furthermore, recent in vitro investigations have shown the down-regulation of key TJ proteins, including ZO-1, occludin, and claudin-1 [60].

In this scenario, the therapeutic potential of c40, a wall fragment derived from a patented and safe-deposit-protected strain of *C. acnes*, alone and conjugated to hyaluronic acid (HAc40) on one of the main pathogenetic mechanisms triggered by *S. aureus* and the down-regulation of tight junctions have been investigated.

The ex vivo experimental model selection based on pig skin biopsies leans on a well-established pertinence to human skin from both histological and physiological viewpoints. From an ethical perspective, pig biopsies were collected as a by-product from slaughterhouses and, in this particular context, do not contradict the Cosmetics Directive. Furthermore, this collection approach can control the animal skin age and the sampling body site [61].

Our results demonstrate that tissue infected with both strains of *S. aureus* and the supernatant containing the α-toxin showed clear damage to the stratum corneum by eosin-hematoxylin staining, confirming the pathogenic action on the epidermis. Evaluation by immunohistochemistry of the expression levels of the tight junction proteins Claudin-1 and ZO-1 deepened the pathogenetic mechanism of *S. aureus* just discussed by revealing a reduced signal in infected tissue compared to control tissue. Surprisingly, all tissues infected with the two *S. aureus* strains and the supernatant containing the toxin but pre-incubated and/or co-incubated with c40 and HAc40 showed a stronger signal comparable to that of the control, suggesting that the wall fragment can interfere with the degradation of tight junction proteins exerted by *S. aureus*.

## 5. Conclusions

To sum up, this study demonstrated that c40 and HAc40 could protect the tissues’ horny layer and tight junction proteins from *S. aureus* infection damage. Indeed, c40 and its functional ingredient HAc40 can be a potential non-pharmacological treatment, acting by counteracting the degradation of the skin barrier caused by *S. aureus* and consequently restoring the natural composition of the skin microbiome. These findings offer numerous avenues for research. Other virulence factors produced by *S. aureus* seem to play a crucial role in the inflammatory process driven by the bacterium (lipoproteins that activate TLR-2 on keratinocytes or the δ-toxin that activates mast cell degranulation) could be taken into account. Finally, interactions between *S. aureus* and the host cell surface, including protein A, clumping factor B, and proteins responsible for fibronectin binding, should be studied in detail.

## Data Availability

The data presented in this study are available on request from the corresponding author.
